# Keloid formation following ear piercing through the transitional zone^☆^

**DOI:** 10.1016/j.abd.2021.08.016

**Published:** 2023-03-28

**Authors:** Ozge Zorlu, Serkan Yazici, Şaduman Balaban Adım

**Affiliations:** a Department of Dermatology and Venereology, Bursa Uludag University School of Medicine, Bursa, Turkey; b Department of Pathology, Bursa Uludag University School of Medicine, Bursa, Turkey

Dear Editor,

Ear piercing is one of the major risk factors for keloid formation. The majority of piercing is performed through the soft tissue of the earlobe only (zone 1). In addition, it may be performed through the ear cartilage (zone 2) or the transitional zone (zone 3) between the ear cartilage and earlobe.^1^ The incidence of complications due to transcartilagenous piercing is approximately 35% because of the avascular nature of auricular cartilage.^1^

There are no studies regarding transitional zone keloids in the literature. We hypothesized that ear piercing through the transitional zone should be assessed as if through the cartilage zone.

We herein present three cases of keloid formation after ear piercing through the transitional zone of the ear. None of our patients had a personal or familial history of keloid or hypertrophic scar formation. The diagnosis was confirmed by histopathological examination for all patients. The clinical features of the patients are presented in Table 1. Patient 1 had a total of four piercings, two in the right earlobe, one in the left earlobe, and one in the right transitional zone. Patient 2 had a total of five piercings, two in the right earlobe, two in the left earlobe, and one in the right transitional zone. All piercings were performed simultaneously in both patients. However, no keloid formation was observed at the earlobe piercing points, in which transcartilagenous piercing did not exist (Fig. 1). Patient 3 had only one piercing in the right transitional zone (Fig. 2). A combination of intralesional corticosteroid administered at 40 mg/mL over intervals of 3‒4 weeks for 16 weeks and cryosurgery were performed. Early recurrence was not observed in any patients during the first year of follow-up.

**Table 1:** Clinical features of the patients.

Patient no	Sex	Age at the ear piercing (yrs)	Time between piercing and keloid formation	Localization of keloid	Other complications due to piercing
1	F	17	<1 y	R, P, zone 3	None
2	F	20	<1 y	R, AP, zone 3	None
3	M	34	<1 y	R, A, zone 3	None

F, Female; M, Male; y, year; yrs, years; R, Right; P, Posterior; A, Anterior; AP; Anterior and Posterior.

**Figure 1:**
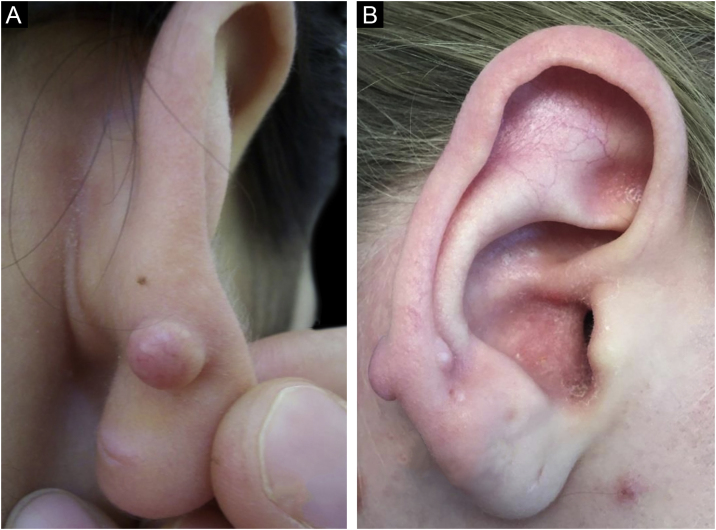
Keloid formation following piercing through the transitional zone in patient 1 (A) and patient 2 (B). There is no keloid formation at the earlobe piercing points in both patients.

**Figure 2:**
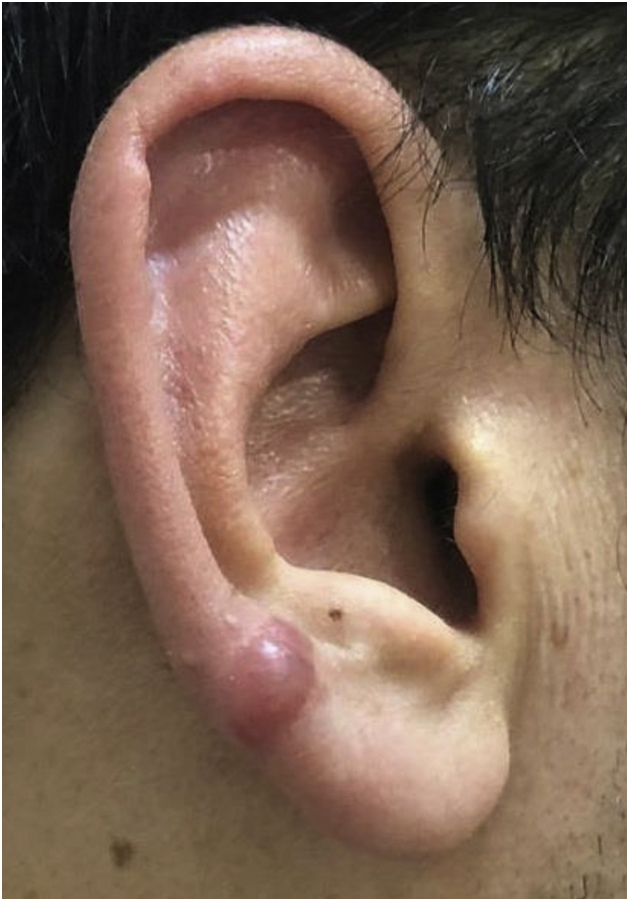
Keloid formation following piercing through the transitional zone in patient 3.

Piercing jewelry material, earring backs, the type of piercing procedure, and complications related to piercing at the time of the procedure may contribute to keloid formation.^2^ None of our patients had a complication related to piercing at the time of the procedure. In our cases, keloid formation was more likely to be associated with a cartilage injury.

Currently, the upregulation of cartilage oligomeric matrix protein (COMP), a noncollagenous extracellular matrix glycoprotein, has been shown in keloidal tissue, suggesting that COMP facilitates keloid formation by accelerating collagen deposition.^3^ In addition, it has been reported that multiple hereditary exostoses, which are characterized by the development of multiple benign osteocartilaginous masses, were found to be a risk factor for keloid formation after surgical excision of osteochondromas representing another association of keloids and chondrocytes.^4^

It was reported that there was no difference regarding piercing-related complications between the earlobe and the cartilaginous part of the ear.^1^, ^5^ This result may be attributed to earlobe piercing being performed more frequently than cartilage or transitional zone piercing.

Assessing ear piercings through the transitional zone as if through the cartilage zone may be more appropriate. Transitional and cartilage zones of the ear may be avoided during ear piercing to prevent the development of keloid formation.

## Financial support

This research did not receive any specific grant from funding agencies in the public, commercial, or not-for-profit sectors.

## Author's contribution

All authors (Ozge Zorlu, Serkan Yazici, Şaduman Balaban Adım) have been actively involved in study conception and planning, critical literature review, data collection, analysis and interpretation, research orientation, preparation and writing of the manuscript, and review of the manuscript. All authors read and approved the final version of the manuscript.

## Conflicts of interest

None declared.
